# Case Report of a Massive Thigh Hematoma after Adductor Canal Block in a Morbidly Obese Woman Anticoagulated with Apixaban

**DOI:** 10.1155/2018/7653202

**Published:** 2018-08-13

**Authors:** Katherine L. Koniuch, Bradley Harris, Michael J. Buys, Adam W. Meier

**Affiliations:** University of Utah Department of Anesthesiology, USA

## Abstract

Hematoma formation after peripheral nerve block placement is a rare event. We report a case of a morbidly obese patient who was anticoagulated with apixaban and developed a massive thigh hematoma after an ultrasound-guided adductor canal block. Despite continuous visualization of the block needle, an unrecognized vascular injury occurred leading to a 14-cm hematoma in the anterolateral thigh. Morbid obesity warrants additional risk consideration when placing nerve blocks in an anticoagulated patient. In addition, early recognition and expert consultation are both important in the management of block-related hematomas.

## 1. Introduction

Peripheral nerve blocks provide effective postoperative analgesia for extremity surgeries [1] and have been associated with few complications [2]. In morbidly obese patients taking anticoagulants near the time of surgery, there may be increased risk of bleeding or hematoma formation which should be considered based on the 2018 American Society of Regional Anesthesia (ASRA) guidelines for Regional Anesthesia in the Patient Receiving Antithrombotic or Thrombolytic Therapy [3]. In this case report, we describe a morbidly obese patient taking apixaban whose postoperative course was complicated by a massive thigh hematoma that developed after an adductor canal block. We detail the factors that may have contributed to this complication, how it could have been avoided, and how the hematoma was managed.

## 2. Case Description

The patient involved provided written consent for reporting of this case.

A 63-year-old woman with medical history of super morbid obesity (BMI 54) and atrial fibrillation for which she was anticoagulated with apixaban presented for an open reduction internal fixation (ORIF) of an ankle fracture. Significant medical history included diabetes mellitus type 2, obstructive sleep apnea, chronic obstructive pulmonary disease, and diastolic heart failure. The patient's last dose of apixaban was 48 hours prior to surgery. Other than moderate anemia (hemoglobin 8.8 g/dL), all laboratory studies, including a coagulation profile, were normal.

Prior to surgery, the patient was offered a sciatic nerve catheter and an adductor canal block as part of a multimodal postoperative analgesia strategy. Because of her many, serious medical conditions, we concluded that a peripheral nerve block offered the best opportunity to provide satisfactory postoperative analgesia. Specifically, we were concerned that the postoperative pain management primarily with opioid medications would pose increased cardiopulmonary risk to the patient. We were careful to explain the risks associated with peripheral nerve blocks, including the risk of bleeding and hematoma formation, and verbal consent was obtained.

The surgery was performed under general anesthesia and her intraoperative course was uncomplicated. Upon arrival to the recovery room, our acute pain service was contacted to evaluate her for peripheral nerve blockade. We positioned the patient in the lateral decubitus position and placed a sciatic nerve catheter. Though technically challenging due to body habitus, this sciatic nerve block was performed successfully and without any complication. The patient was then positioned supine for the adductor canal block. The leg was externally rotated and the knee slightly flexed for optimal positioning. A high-frequency linear array ultrasound transducer was applied to the mid-thigh in short-axis and the adductor canal was identified. Imaging was again challenging given the patient's habitus, but with firm compression of the ultrasound transducer, the important anatomical structures were clearly identified. The superficial femoral artery (SFA) was visualized dorsal to the sartorius muscle and a hyperechoic structure anterolateral to the artery was identified as the adductor canal and saphenous nerve [4, 5]. The skin adjacent to the probe was cleansed with a chlorhexidine and alcohol solution. A 20-gauge × 4-inch beveled, echogenic needle was inserted using an in-plane technique. The needle was visualized continuously as it coursed between the vastus medialis and sartorius toward the adductor canal. The needle was positioned lateral to the SFA within the canal and a bolus of 20 ml of 0.25% bupivacaine with 5 mcg/ml epinephrine was administered with negative heme aspiration checks after every 5 ml injection. Spread of local anesthetic within the adductor canal was clearly observed under ultrasound visualization. There was no evidence of intravascular injection of epinephrine while monitoring the patient. Upon needle withdrawal, brisk bleeding was noted at the skin insertion site but with direct manual pressure for approximately 60 seconds, bleeding ceased completely.

**Figure 1:**
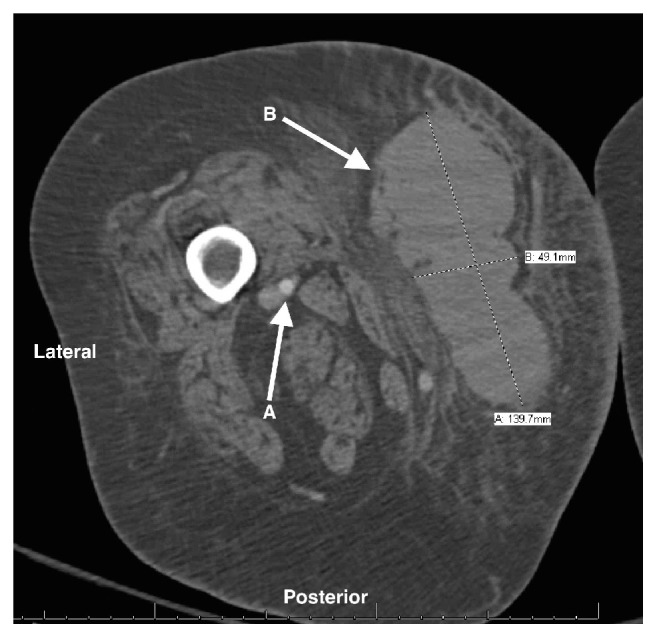
CT of the patient's right thigh showing the superficial femoral artery (A) and 5 × 14 cm hematoma (B).

Shortly after the blocks were performed, the patient reported complete resolution of her ankle pain and was transferred to her hospital room. Approximately 6 hours after surgery, the patient reported new anterior thigh pain on the operative leg, which was treated by her nurse with intravenous hydromorphone. Roughly 13 hours after surgery, the patient's nurse finally contacted the orthopedic surgery team due to unmanageable mid-thigh pain. The orthopedics team initially believed the pain was due to tourniquet compression pain which occurred during surgery. Upon further physical examination, a hematoma was noted in the anterolateral mid-thigh. Vital signs were within normal ranges and distal pulses were intact. A CT scan with contrast was ordered and revealed a 14-cm hematoma in the right thigh (Figure 1). Lab studies showed a drop in hemoglobin from 8.8 g/dL preoperatively to 6.9 g/dL the morning of postoperative day (POD) 1. Coagulation studies at that time were within normal limits including partial thromboplastin time, prothrombin time, and international normalized ration, as well as platelet count. The patient's primary medicine service transfused 1 unit of packed red blood cells, which improved her hemoglobin to 7.6 g/dL.

**Figure 2:**
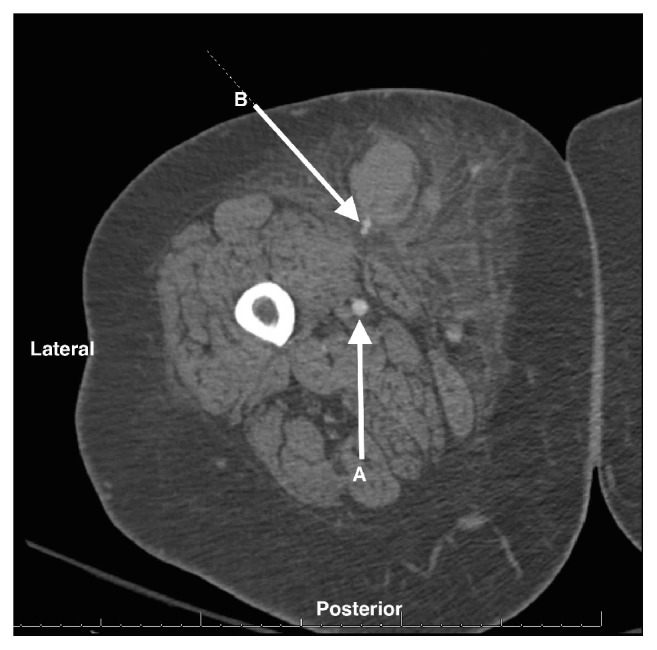
CT angiogram of the patient's right thigh showing the superficial femoral artery (A) and active extravasation from a small branch of the SFA (B).

Interventional radiology was consulted on the morning of POD 1 for management recommendations for the hematoma. A CT angiogram was performed revealing active extravasation from a small superficial branch of the SFA (Figure 2). Embolization with coil and gel foam was performed and compressive dressings were used to apply direct pressure. Throughout the day, the patient's hemoglobin remained stable and the hematoma showed no evidence of further expansion.

On POD 2, further consultation from the vascular surgery and interventional radiology teams was sought for possible hematoma evacuation versus drain placement given the massive size. Vascular surgery recommended conservative management with application of direct pressure. Interventional radiology, however, recommended placement of a drain within the hematoma. A pigtail catheter was placed and fluid cultures were obtained. Minimal output from the drain was observed, so beginning on POD 3, tissue plasminogen activator (TPA) was administered through the catheter daily to facilitate hematoma drainage. Follow-up ultrasound on POD 6 showed a persistent hematoma despite TPA administration. Hematoma cultures resulted negative for infection. On POD 7, she was discharged to a rehabilitation facility with the drain in place and care team instructions to flush 5-10 ml saline twice daily until evaluation with interventional radiology one week later.

On POD 14, the drain was inadvertently pulled out requiring a return visit to interventional radiology and drain replacement. On POD 19, the hematoma cavity had decreased to an acceptable size so the drain was removed. One week later, on POD 26, she returned to interventional radiology due to increased pain and swelling at the hematoma site. A recurrent fluid collection was noted on ultrasound examination so the drain was replaced a third time and aspirated fluid was sent for culture, which grew staphylococcus aureus. She was treated for hematoma superinfection with a five-day course of levofloxacin and when she returned two weeks later, the abscess had resolved and the drain was removed.

## 3. Discussion

This case demonstrates important points regarding regional anesthesia in an anticoagulated, morbidly obese patient. First, morbid obesity represents an additional risk factor when considering a superficial peripheral nerve block on an anticoagulated patient. Second, there is a paucity of case reports detailing the complication of hematoma formation due to a peripheral nerve block. Third, the subsequent management of the hematoma after peripheral nerve block is not well reported in the literature and presents a considerable challenge, especially in patients with multiple comorbidities.

Peripheral nerve blocks can provide excellent analgesia for patients having ankle surgery [6]. In a morbidly obese patient, a nerve block may significantly reduce the need for opioid administration [7, 8]. This can provide great benefit for patients at risk for postoperative opioid-induced hypoventilation. When considering the risks and benefits of a nerve block for our patient, opioid reduction was a clear benefit. Throughout her hospital course, she never received opioids for ankle pain while nerve blocks were in effect. Thus we believe there was a clear analgesic benefit to regional anesthesia.

For this patient, however, the risk of bleeding due to her anticoagulation status was also concerning. Although vascular injuries resulting from peripheral nerve blocks are rare complications [2, 7, 9, 10], this case demonstrates the significant morbidity that may occur. The recently updated (2018) ASRA guidelines recommend “management based on site compressibility, vascularity, and consequences of bleeding.” [3] While an adductor canal block is considered to be superficial, we feel it is more appropriately categorized as a deep block in a morbidly obese patient because compressibility is more difficult if inadvertent vascular puncture were to occur. In addition, given the potential for massive bleeding into the thigh, additional consideration should be made for procedures on the upper leg. Anticoagulation recommendations for deep peripheral blocks are the same as neuraxial techniques—the ASRA guidelines recommend waiting 72 hours after the last dose of apixaban for block placement [3]. Although it had only been 48 hours since our patient's last dose, we felt the benefit of opioid reduction would outweigh the risk of vascular injury or significant bleeding.

In this case, the anesthesiologist performing the adductor canal block was an experienced regional anesthesiology fellow who had performed adductor canal blocks in this same manner many times before. A fellowship-trained regional anesthesiologist supervised the nerve block placement. Direct visualization of the needle and of the neurovascular structures was uninterrupted. We believe the vascular injury likely occurred when the needle passed through the superficial branch of SFA as the ultrasound probe was compressing it. This small branching artery was never recognized on ultrasound imaging. Although the vascular injury may not have been easily avoided regardless of the patient's coagulation status, waiting an additional 24 hours to perform the block (72 hours after her last apixaban dose) may have decreased the extent of hematoma formation. While this may have made no difference in her clinical outcome, perhaps it can be used to better guide risk versus benefit discussions in morbidly obese patients who are anticoagulated before receiving a peripheral nerve block.

Another factor that contributed to the significant morbidity in this case was the management of the hematoma after it was recognized. The patient underwent multiple procedures to drain the hematoma and required return to the hospital for drain replacement, and ultimately her course was complicated by an infection of the hematoma cavity. Despite a thorough literature search, we were unable to find clear recommendations as to the management of extremity hematomas. We believe that a more conservative approach of close monitoring instead of drain placement would have resulted in an acceptable outcome without additional procedures, increased cost, a prolonged hospital stay, and increased risk of infection.
